# An Aptamer-Based Biosensor for the Azole Class of Antifungal Drugs

**DOI:** 10.1128/mSphere.00274-17

**Published:** 2017-08-23

**Authors:** Gregory R. Wiedman, Yanan Zhao, Arkady Mustaev, Jinglei Ping, Ramya Vishnubhotla, A. T. Charlie Johnson, David S. Perlin

**Affiliations:** a Public Health Research Institute, New Jersey Medical School-Rutgers Biomedical and Health Sciences, Newark, New Jersey, USA; b Department of Physics and Astronomy, University of Pennsylvania, Philadelphia, Pennsylvania, USA; JMI Laboratories

**Keywords:** antifungals, aptamers, graphene, biosensors

## Abstract

This technical report describes the development of an aptamer for sensing azole antifungal drugs during therapeutic drug monitoring. Modified synthetic evolution of ligands through exponential enrichment (SELEX) was used to discover a DNA aptamer recognizing azole class antifungal drugs. This aptamer undergoes a secondary structural change upon binding to its target molecule, as shown through fluorescence anisotropy-based binding measurements. Experiments using circular dichroism spectroscopy revealed a unique G-quadruplex structure that was essential and specific for binding to the azole antifungal target. Aptamer-functionalized graphene field effect transistor (GFET) devices were created and used to measure the strength of binding of azole antifungals to this surface. In total, this aptamer and the supporting sensing platform provide a valuable tool for therapeutic drug monitoring of patients with invasive fungal infections.

**IMPORTANCE** We have developed the first aptamer directed toward the azole class of antifungal drugs and a functional biosensor for these drugs. This aptamer has a unique secondary structure that allows it to bind to highly hydrophobic drugs. The aptamer works as a capture component of a graphene field effect transistor device. These devices can provide a quick and easy assay for determining drug concentrations. These will be useful for therapeutic drug monitoring of azole antifungal drugs, which is necessary to deal with the complex drug dosage profiles.

## Abstract (precis)

We have developed the first aptamer directed toward the azole class of antifungal drugs and a functional biosensor for these drugs. This aptamer has a unique secondary structure that allows it to bind to highly hydrophobic drugs. The aptamer works as a capture component of a graphene field effect transistor device. These devices can provide a quick and easy assay for determining drug concentrations. These will be useful for therapeutic drug monitoring of azole antifungal drugs, which is necessary to deal with the complex drug dosage profiles.

## INTRODUCTION

Understanding a drug’s pharmacokinetics is crucial to safely and effectively treating patients. Unfortunately, drug levels in patients can vary significantly, and the factors contributing to this variability are frequently misunderstood. For some critically ill patients, it is essential to gauge levels of a drug in real time. The best therapeutic management can be achieved by maintaining a therapeutic level in a patient’s bloodstream and by optimizing individual dosage regimens. These analyses generally rely upon trough and peak monitoring and real-time kinetic drug modeling. For this reason, therapeutic drug monitoring (TDM) of some drugs is a critical component of successful therapy (1). It is particularly important to monitor drugs with narrow therapeutic ranges, marked pharmacokinetic variability, target concentrations that are difficult to monitor, and known to cause adverse events.

The azole antifungal drugs posaconazole, fluconazole, voriconazole, and itraconazole are an important class of lanosterol 14α-demethylase enzyme-inhibiting molecules (2), which compromise fungal cell membranes by preventing the synthesis of the key component ergosterol (3). A number of these drugs are highly hydrophobic, which creates analytical challenges. Furthermore, because of their hydrophobic nature it is difficult to know how much of the drug is freely available in the blood at any given time. Wide variances in the pharmacokinetics of critically ill patients have been observed for triazole drugs like voriconazole and posaconazole, which has resulted in a need for TDM (4). Furthermore, posaconazole and voriconazole have been shown to have drastically different bioavailabilities depending on how they are administered and if they are coadministered with other drugs (5, 6). Therapeutic drug monitoring in conjunction with antifungal therapy has been shown to promote a more favorable outcome than in non-TDM groups (7). Unfortunately, TDM requires blood to be drawn from patients and then drug levels in blood to be evaluated by analytical instrumentation at some later point in time. Analytical techniques such as liquid chromatography (LC) and mass spectrometry (MS) require skilled staff and resources that are not found in all hospitals (8). These barriers become especially difficult to overcome when treating patients in community hospitals, at home, or in resource-limited settings. Effective methods for sensing small drug molecules in blood samples would make it easier to determine drug concentrations.

Any effective TDM method requires a way to capture the drug target from a patient sample. Antibodies provide specificity and sensitivity as a capture probe, but they are typically unstable over a wide range of assay conditions. As a more robust alternative, oligonucleotide-based aptamer capture probes were developed here as a stable and selective capture molecule for small-molecule drugs. Oligonucloetide aptamers can bind to a wide variety of target molecules with high affinity. Such oligonucleotides (i.e., DNA and RNA libraries of 10^14^ to 10^16^ molecules) can be quickly synthesized and screened using *in vitro* synthetic evolution of ligands through exponential enrichment (SELEX) methods (9, 10).

In this report, azole-specific aptamers were created by using a modified SELEX method to screen a library of more than 10^14^ DNA sequences. Furthermore, graphene field effect transistors (GFETs) were developed as a biosensing platform for detection of azole antifungal drugs with these aptamers. These devices represent a newly emerging type of biosensor that relies on electronic measurements of the transistor itself rather than the flow of an electrolyte solution, binding of antibodies, or fluorescence labeling. Taken together, these results provide a possible path forward for development of an azole antifungal sensing device with potential broader downstream capability of improving therapeutic drug monitoring of small-molecule drugs.

## RESULTS

### SELEX process results.

**FIG 1:**
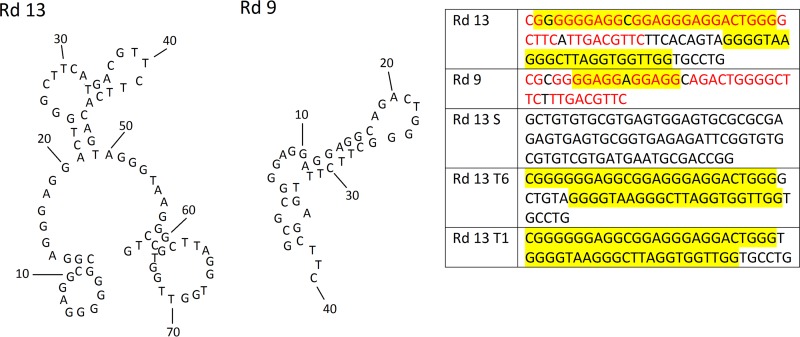
Possible stem-loop structures of Rd 13 and Rd 9 along with the control sequences.

**FIG 2:**
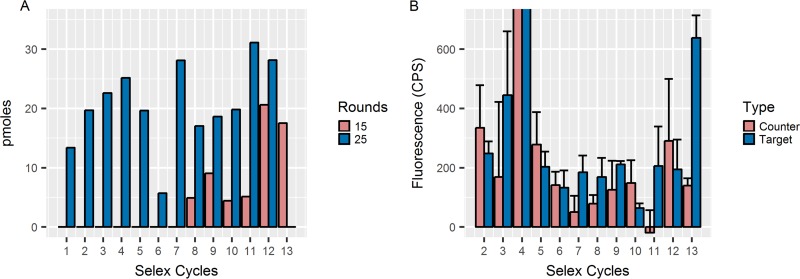
(A) After rounds 5 and 6, the PCR output decreased due to increased pressure. The output increased in later rounds, and amplification can even be seen after only 15 cycles of PCR as opposed to 25 cycles. In panel B, the fluorescence intensity of the Oligreen dye from aptamer samples incubated with posaconazole (target)-labeled beads increased relative to control beads as binders were enriched.

**FIG 3:**
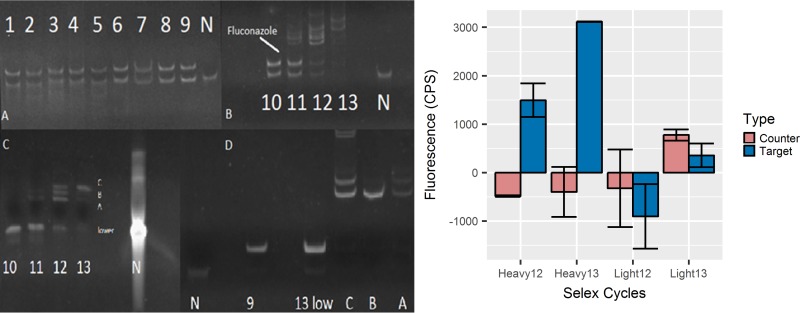
After the addition of fluconazole, there were bands that were higher in molecular weight than the starting library, N40 (lane N), and the original sequences. A denaturing polyacrylamide gel (panel C) highlights the fact that these bands were, in fact, aptamers with unique molecular weights and not aggregates of the smaller original library sequence N40. The relative binding capabilities of the heavy and light bands were different from those of the control (counter-SELEX) and target (posaconazole) labeled beads, respectively. Light bands from rounds 12 and 13 showed little preference for either type of beads. The heavy bands, however, bound significantly better to the target labeled beads than the control beads. Negative values occurred when emission at 525 nm was below that at 505 nm. Lanes C, B, and A in panel D correspond to the separated bands C, B, and A in panel C.

Azole-binding aptamers were generated from a random 40-mer library using a modified SELEX process (Fig. 1). PCR output and Oligreen dye intensity were used to track the enrichment of posaconazole binders. The output of the PCR experiments for each selection round was plotted after both 15 and 25 cycles of PCR amplification. In addition, the intensity of Oligreen dye on DNA-containing counter-SELEX and SELEX (target) beads (Fig. 2A and B) was used to track enrichment. During rounds of increased pressure, such as rounds 5 and 6, the total PCR output initially decreased but then recovered over the next rounds. This pattern was most evident past round 10, when the beads were first washed with the competitive molecule fluconazole. This wash allowed for weakly binding molecules to be eluted from the sample. Denaturing polyacrylamide gels were used to assess whether or not the aptamer’s molecular weight changed during SELEX. Each round displays two bands, which are a result of leftover double-stranded DNA not digested by λ exonuclease (Fig. 3A). The single-strand bands from rounds 1 to 10 are the same size as the single-strand control library. The larger bands from rounds 11, 12, and 13 are distinct from the library band even when denatured (Fig. 3C). Each band was separated as described in the Materials and Methods section and further analyzed. This study also included several control sequences to further investigate the importance of the structure of the round 13 aptamer (Rd 13): Rd 13 Scrambled (S), Rd 13 T6, and Rd 13 T1 were created with various middle lengths. Rd 13 Scrambled was carefully reordered from the original sequence to fully prevent the formation of G-quadruplex structures. The two other sequences maintained the G-quadruplex regions but with two different distances between them.

### Selection of best sequence.

After separation, the “heavy” and “light” bands were analyzed separately for their binding capabilities. The light bands of rounds 12 and 13, those the same length as the library, do not bind to the target (Fig. 3). The heavy bands, however, bound significantly better to the posaconazole-labeled beads versus the control beads and were used for further studies. The heavy bands from round 13 as well as the sample from round 9 were sequenced.

The sequence for Rd 9 is the same size, 40 bases, as the original library. The sequence for Rd 13 is almost double that size at 79 bases. A significant portion of the Rd 9 sequence (in red in Fig. 1) is found in the Rd 13 sequence. Additionally, QGRS mapping was used to predict the existence of G-quadruplex structure in these sequences (11). The Rd 9 sequence contains one predicted G-quadruplex stretch, highlighted in Fig. 1. Interestingly, the Rd 13 sequence contains two regions of predicted G-quadruplex structure. Stem-loop structure-predicting software was used to map the room temperature (298-K) structures of these aptamers (12). These predictions show that while Rd 9 forms a stem-loop structure with a single arm, Rd 13 has two separate arms. These features became a major focus of the further study of these aptamers.

### Binding affinity of azole drug aptamers.

**FIG 4:**
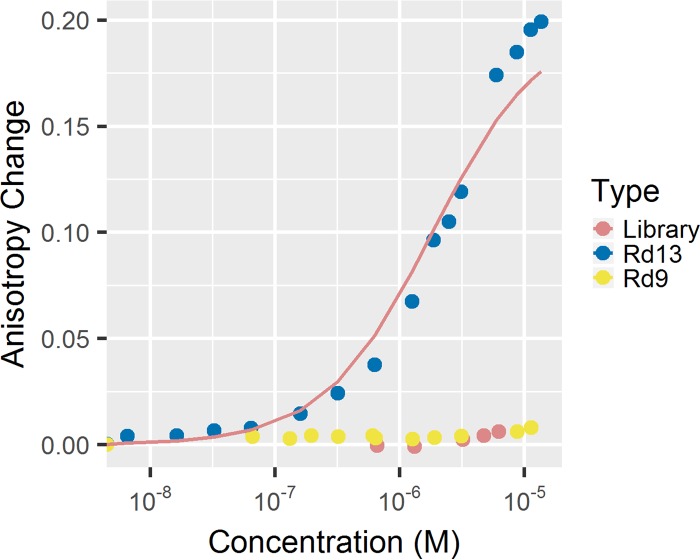
PosBD anisotropy changed when the aptamer was titrated into 100 pmol of PosBD per 125 μl. (Example traces are shown.) The anisotropy changed neither with the earlier round (round 9) nor with the control library.

**FIG 5:**
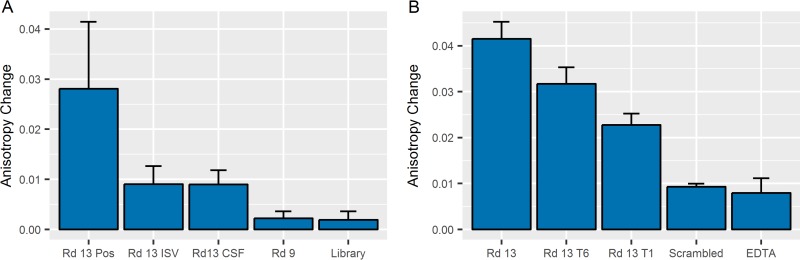
One hundred picomoles of aptamer (Rd 13, Rd 9, or the library) was titrated into 100 pmol of various BODIPY-labeled drugs (posaconazole [Pos], isavuconazole [ISV], and caspofungin [CSF]) (A). Titration of Rd 13 into PosBD caused a significant change in anisotropy. Titration of Rd 13 into other BODIPY-labeled drugs or Rd 9/library into PosBD caused little changes in anisotropy. These data suggested that Rd 13 binds best to posaconazole. Aptamers of different lengths were titrated into 100 pmol of PosBD (B). The full-length Rd 13 aptamer caused the greatest change in anisotropy, and the truncated and scrambled versions showed decreased amounts of anisotropy change. The scrambled version only caused a change proportional to that caused by a chelator, EDTA.

Fluorescence anisotropy experiments were used to determine the dissociation constants (*K*_*d*_s) for the posaconazole-aptamer complex (11, 12). These experiments measured the ability of a boron-dipyrromethene (BODIPY)-labeled posaconazole (PosBD) to rotate in solution. Inhibited rotation, due to aptamer binding, was detected as a change in anisotropy. In these experiments, only the Rd 13 aptamer caused an increase in anisotropy from titrations of a constant PosBD concentration with increasing amounts of DNA aptamers (Fig. 4). The control library and Rd 9 fail to bind to the PosBD. It should be noted that the control library contains the primer sequences, bringing the total length to 86 nucleotides. This fact suggests that the difference in molecular weights between Rd 13 and Rd 9 is not the sole reason for the difference in anisotropy. The dissociation constant when fitting to the fraction bound (*F*_bound_) is 2.7 ± 1.2 μM. The overall dissociation constant for PosBD might be weaker than that of posaconazole given the fact that PosBD was not used for SELEX. The differences in anisotropy changes were further used to probe the specificity of the aptamer for the target. Specificity was interrogated with respect to two other BODIPY-labeled molecules: isavuconazole (ISV), which is chemically similar to posaconazole, and caspofungin (CSF), which is chemically dissimilar since it is an echinocandin class drug. Titration of 100 pmol of Rd 13 aptamer into a 125-μl solution containing 100 pmol of PosBD causes a greater anisotropy change than titration into 100 pmol of BODIPY-labeled ISV or CSF (Fig. 5). Titration of Rd 9 or the library into 100 pmol of PosBD causes little to no change in anisotropy. The truncated versions T6 and T1 cause less of an anisotropy change, although Rd 13 T6 is not significantly different (Fig. 5B). The Rd 13 Scrambled aptamer did not cause a significant anisotropy change compared to a control such as EDTA and hence did not bind to PosBD. These results indicated that the G-quadruplex structure is necessary for binding.

### Binding competition assay.

**FIG 6:**
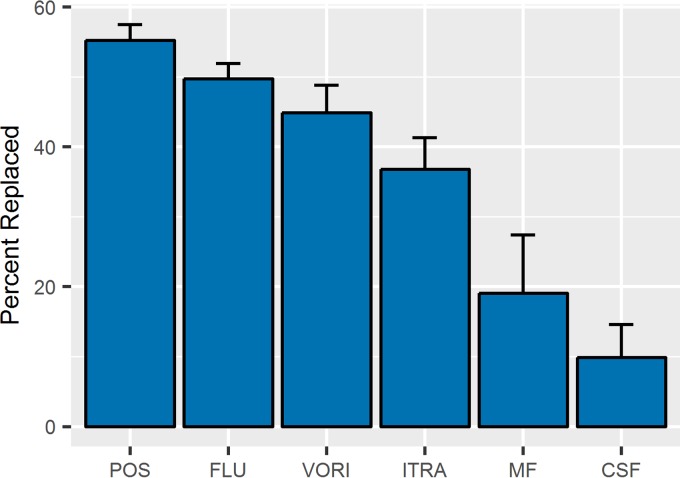
After initial PosBD incubation, the aptamer was heated at 70°C in the presence of posaconazole (Pos), fluconazole (Flu), itraconazole (Itra), voriconizole (Vori), micofungin (MF), or caspofungin (CSF) and then cooled on ice. The percentage replaced was calculated as a function of the loss in anisotropy.

The aptamers developed in this study bind to azole class antifungal drug targets, specifically to those with the exposed terminal azole group like posaconazole. High specificity is important for downstream diagnostic devices to prevent false-positive readings. The anisotropy experiments were modified slightly to develop a competitive assay to further probe specificity. This experiment showed the relative abilities of various drugs to replace PosBD in the aptamer complex (Fig. 6). As expected, posaconazole displaces the greatest amount of PosBD. The related drugs fluconazole and itraconazole replace fewer PosBD molecules. The chemically distinct echinocandin antifungal drugs micafungin and caspofungin had little effect. Of the azole drugs, itraconazole is the most hydrophobic. The smaller amount of PosBD replacement with itraconazole versus posaconazole suggests that binding is not solely driven by hydrophobic effects. Secondary structure plays a large part in the binding of azole targets to these aptamers.

### CD spectroscopy and secondary structure analysis.

**FIG 7:**
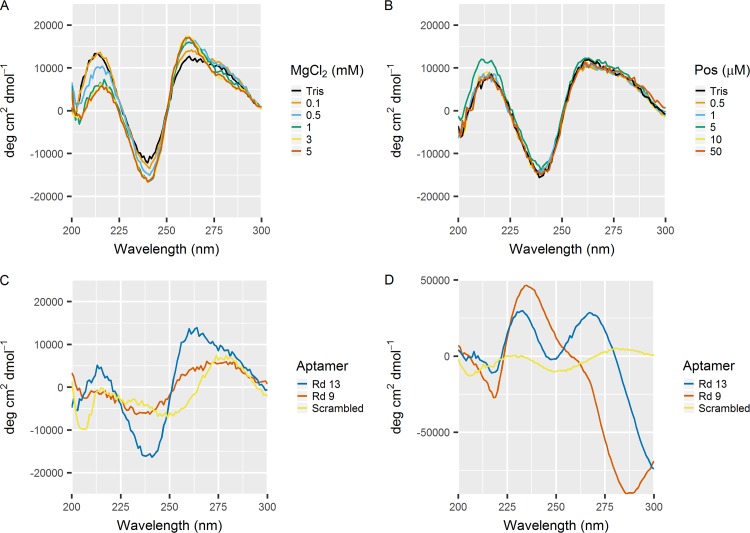
Addition of magnesium chloride to the Rd 13 aptamer enhances G-qudruplex folding (A), but addition of posazonazole alone does not (B). This is seen in an increase in the maximum at 260 nm and a decrease in the minimum at 240 nm. Both Rd 13 and Rd 9 exhibit G-quadruplex structure in 0.2 mM magnesium chloride salt (C). The structure changes drastically in the presence of magnesium chloride and 100 μM posaconazole (D). The Rd 13 Scrambled aptamer does not change, but Rd 9 now contains a peak of 230 nm and a minimum at 280 nm and Rd 13 contains two peaks at 230 nm and 260 nm with a new minimum peak above 300 nm.

Circular dichroism (CD) spectroscopy experiments were conducted to probe the folding of these aptamers in the presence of salts and posaconazole. CD spectroscopy is a technique that is widely used in biophysics to predict secondary structures of biomolecules (13). Molecules such as nucleotides and proteins can contain structures that will interact differently with left- and right-polarized light, which can be detected by CD spectroscopy. Secondary structure controls the complex formation of aptamers and target molecules. As these aptamers all contained multiple stretches of guanine residues, there is a high possibility that they form G-quadruplex structures. The CD spectra of Rd13 are characteristic of G-quadruplex folded DNA, with a maximum at 260 nm and a minimum at 240 nm (Fig. 7A) (14, 15). Addition of magnesium chloride to the solution both increases the signal at 260 nm and decreases the signal at 240 nm. This suggests that the aptamer forms a G-quadruplex structure in low-salt buffer, which is slightly enhanced with the addition of salts. In contrast, the signal is not altered significantly by adding posaconazole in the absence of divalent salts (Fig. 7B). The most drastic change occurs when the aptamers are exposed to a combination of both posaconazole and salts. The aptamers Rd 9 and Rd 13 show similar CD signals in 0.2 mM magnesium chloride. A G-quadruplex structure formed with Rd 9 and Rd 13 but not with Rd 13 Scrambled (Fig. 7C). When 100 μM posaconazole was added, there was a change in the CD signal for Rd 9 and Rd 13. With posaconazole, the spectrum for Rd 9 changes to contain a maximum at 230 nm and a drastic minimum at 280 nm (Fig. 7D). The spectra for Rd 13 in magnesium chloride with posaconazole contain two maxima at about 230 and 270 nm with a minimum above 300 nm. The Rd 13 Scrambled sequence does not undergo any further change in secondary structure. The G-quadruplex structure of Rd 13 forms in the presence of salt, and this structure then changes when the target is added.

### GFETs.

**FIG 8:**
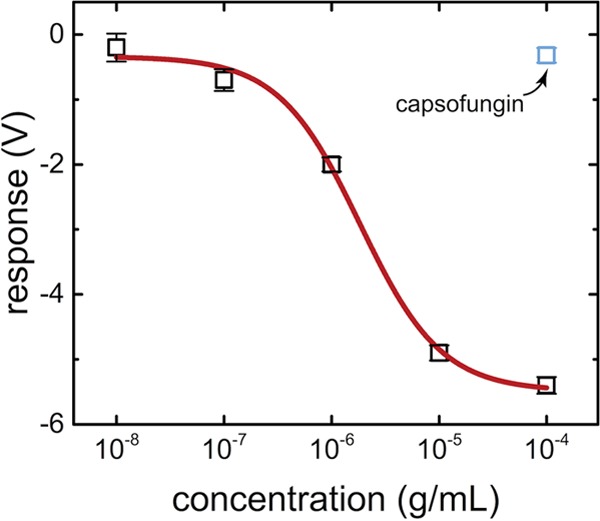
The aptamer-based GFET devices showed a detectable threshold Dirac voltage shift between 0.1 mg/ml and 0.1 μg/ml with posaconazole (Pos) and none with CSF. The red curve indicates a fit to a Hill-Langmuir equation. The fit values, especially the low microgram-per-milliliter range are therapeutically relevant concentrations of azole class antifungal drugs.

Graphene field effect transistors (GFETs) are a robust platform for detecting the binding of small molecules to a surface (16). As a transistor, GFETs allow for the flow of charge between a gate and a source over a single sheet of carbon atoms. This sheet is extremely sensitive to changes or binding above it, seen as a change in the Dirac voltage needed for charge to flow. When combined with GFET devices, these aptamers act in an induced-fit manner, which allows them to function as a small-molecule capture arm on a supported surface. GFET devices functionalized with amino-Rd 13 were used to measure the posaconazole concentration (Fig. 8). Posaconazole was diluted from dimethyl sulfoxide (DMSO) into SELEX buffer as described above. The sensor output signal was taken to be the Dirac voltage shift, measured relative to the shift induced upon exposure to pure buffer. As concentrations of posaconazole were increased from 0.01 μg/ml to 100 μg/ml, the relative Dirac voltage shift increased to upwards of −6 V. The variation of the relative Dirac voltage shift with concentration was well fit by a model based on the Langmuir-Hill theory of equilibrium binding, where the dissociation constant of the aptamer is a fitting parameter (17). The best-fit value of 1.8 ± 0.5 μg/ml (2.6 ± 0.7 μM) is in good agreement with the value of 2.7 ± 1.2 μM derived from the anisotropy assay. In a negative-control experiment, treatment with the echinocandin drug caspofungin produced a negligible shift in the gate threshold voltage, providing strong evidence that the sensor response reflects specific binding of the target to the aptamer probe. The observed values of the relative Dirac voltage shift are in a range similar to that of a similarly designed aptamer-based GFET biosensor biosensing for an HIV drug (26). These data suggest that Rd 13 aptamer chemically attached to the GFET surface binds posaconazole in a similar fashion to free Rd 13 aptamer in solution.

## DISCUSSION

Therapeutic drug monitoring requires a method of capturing molecules and separating them from a sample for analysis. This report highlights the development of an azole drug-capturing oligonucleotide using the SELEX process and the discovery of a unique structure that allows it to bind azole antifungal drugs. Circular dichroism spectrophotometry showed that this oligonucleotide works as a scaffold with two sections of G-quadruplex folds. Large protein target aptamers have been made before using two separate G-quadruplexes linked chemically to bind at separate sites (18). These types of folds rarely interacted with smaller, hydrophobic molecules due to the highly charged nature of single-stranded DNA (ssDNA). The larger Rd13 aptamer was likely generated as a result of the interaction between single G-quadruplex-containing aptamers. When azole drugs bind to these aptamers, a structural change occurs. The CD spectra of these changes are similar to CD spectra for B→Z DNA transitions (19, 20). This type of dual-G-quadruplex aptamer proved ideal for capturing small hydrophobic molecules. The anisotropy binding experiments show that the poly(G) region is essential for target binding. When bound to the surface of a graphene field effect transistor, the aptamer works as a capture arm. This arm collects posaconazole from the sample, which leads to a change in the GFET gate voltage. The azole “aptasensor” adds to the list of other aptamer-based sensing devices (21, 22). Unlike these other devices, however, GFET aptamer biosensors have the potential to function without the need for secondary antibodies, fluorophores, or electrochemical mediators (23). The versatility of the oligonucleotide-based biosensor opens the door to numerous different applications.

Taken together, the unique structure and binding properties of the oligonucleotide provided with the sensitivity of graphene field effect transistors could prove useful for therapeutic drug monitoring. Posaconazole and the other azole class antifungal drugs exhibit strong hydrophobicity and protein binding. Despite this fact, this aptamer binds specifically to azole drugs. There are other aptamers directed toward clinically interesting drugs, as well such as the aminoglycoside antibiotics and antiretroviral drugs among others (24, 25). The work presented here provides the first basic step toward effective therapeutic drug monitoring: a method of capturing and sensing the drug. These other aptamers could also be utilized as capture arms for a graphene-based sensing platform. The next step will involve testing patient samples and validating their usefulness in clinic. In the near future, aptamer-based GFET biosensors could be mass produced for a fraction of the cost of other methods such as liquid chromatography-mass spectrometry (LC-MS). These tests can be performed in a manner of minutes, negating the need for culture-based methods, which can take upwards of 24 to 48 h. Such devices will allow clinicians to quickly assess azole concentrations in a patient’s blood and provide them with the additional care that they need.

## MATERIALS AND METHODS

The N40 DNA aptamer library was purchased from TriLink BioTechnologies, Inc. (Ronkonkoma, NY). Other oligonucleotides, including amino-functionalized oligonucleotides, were synthesized by IDT (Coralville, IA), Sigma-Aldrich (St. Louis, MO), and Biosearch Technologies (Novato, CA). Carboxyl Dynabeads (14305D), the Oligreen single-stranded DNA (ssDNA) assay kit, and BODIPY fluorescent dye were purchased from Life Technologies, Inc. (Carlsbad, CA). Streptavidin-conjugated magnetic beads and λ exonuclease were purchased from New England Biolabs (Boston, MA). Azole drugs were purchased from Santa Cruz Biotechnologies (Dallas, TX). All other reagents and solvents were purchased from Thermo Fisher Scientific (Waltham, MA). Graphene devices were fabricated in house using methods described in previous work.

### SELEX process.

The aptamer library from TriLink BioTechnologies was prepared by dissolving 1 nmol of DNA in 100 μl of SELEX buffer (140 mM sodium chloride, 2 mM potassium chloride, 5 mM magnesium chloride, 2 mM calcium chloride, 0.05% Tween in 20 mM pH 7.4 Tris buffer). The library was heated at 94°C for 3 min, placed on ice for 5 min, and then incubated at room temperature (25°C) for 5 min. Next, the DNA library was incubated for 1 h at 50°C and 2 h at room temperature and then in later rounds for 10 min at 50°C and 20 min at room temperature. For the first round, the library was incubated with 1 mg of unlabeled carboxyl Dynabeads (counter-SELEX). The beads were carefully concentrated using a magnet. The library was then incubated with 1 mg of posaconazole-labeled beads for 1 h at 50°C and 2 h at room temperature. The beads were washed 3 times with 100 μl SELEX buffer and once with 100 μl Millipore water incubated with 1 nmol posaconazole in 20 µl of water with 0.01% DMSO for 1 h. The recovered DNA was purified using a Zymo Research DNA preparation column. Recovered DNA was amplified under two PCR conditions. First, the 20 μl of DNA was amplified using a TaKaRa rTaq DNA polymerase enzyme. The mixture contained 2.5 μl (10 μM) forward primer, 2.5 μl (10 μM) phosphorylated reverse primer, 5 μl (2.5 mM) deoxynucleoside triphosphates (dNTPs), 5 μl 10× MgCl_2_ buffer, 0.5 μl (2.5 U) rTaq enzyme, 20 μl DNA, and 14.5 μl Millipore water. PCR was performed under the following conditions: an initial round at 94°C for 5 min and then 15 cycles of 94°C for 30 s, 50°C for 30 s, and 72°C for 1 min followed by a final 5 min of extension at 72°C. This sample was then treated as is with 1 μl (5 U) λ exonuclease and incubated at 37°C for 30 min. This mixture was purified using a Zymo column, and the output was checked for absorbance at 260 nm using a Nanodrop spectrophotometer. If the yield was less than 1 pmol, an additional PCR was conducted as described above but substituting 10 cycles for the 15 cycles. Further rounds of SELEX included the following modifications: for rounds 2 through 5, 200 pmol beads was used instead of 1 nmol. After round 5, incubation times were decreased to 30 min for counter-SELEX and then 10 min at 50°C and 20 min at room temperature. After round 6, the bead capacity was decreased to 20 pmol. In rounds 11 to 13, beads were incubated for 1 h with 1 μl of 1 M fluconazole in 20 μl, first as an additional competitive wash, before washing with posaconazole.

### SELEX result tracking.

In each round, 1 μl of DNA-incubated control beads and 1 μl of DNA-incubated posaconazole-labeled beads were saved after washing with water but before posaconazole elution. The DNA content was assessed using a 1:800 solution of Oligreen dye in 20 mM Tris buffer with 2 mM EDTA at pH 7.5, and the samples were analyzed using a Photon Technology International (PTI) fluorometer. PCR output was measured using a Nanodrop spectrophotometer. DNA size was investigated by running an 8% polyacrylamide gel, and denaturing gels were run using an 8% polyacrylamide gel with 8 M urea in SDS buffer after loading DNA treated with formamide at 100°C. Sequencing of various rounds was performed by Macrogen (Rockville, MD) to determine the sequence for that round.

### Fluorescence binding experiments.

Purified bands and synthesized sequences were prepared by taking 2 pmol and dissolving them in 100 μl SELEX buffer. These solutions were heated at 94°C for 3 min, on ice for 5 min, and at room temperature for 5 min. The samples were incubated with 20 pmol posaconazole-labeled and unlabeled beads at 50°C for 10 min and room temperature for 20 min. The samples were then washed 2 times with water, and 125 μl of a 1:800 dilution of Oligreen dye was added. Samples were then heated at 94°C for 3 min, the beads were concentrated, and the supernatant was collected. Samples were excited at 480 nm with emission scanning from 500 to 550 nm. The fluorescence was recorded as counts per second at 520 nm minus the counts per second at 505 nm.

### Fluorescence anisotropy binding experiments.

Fluorescence anisotropy experiments were conducted using a PTI fluorometer with fluorescence polarizers. One hundred picomoles of BODIPY-labeled posaconazole (PosBD) was added from DMSO (1 μl) to 125 μl of modified SELEX buffer (140 mM sodium chloride, 2 mM potassium chloride, 5 mM magnesium chloride, 2 mM calcium chloride in 20 mM Tris buffer, pH 7.4) (12). Fluorescence anisotropy experiments were recorded using a polarizer system, and the G-factor was calculated manually for each run but consistently fell within 0.44 to 0.45. Anisotropy measurements were recorded first for 2 min. After the initial 2 min, aliquots of aptamers from 1 to 2,000 pmol were added, and samples were equilibrated for 5 min. The value of the anisotropy was taken to be the average anisotropy of the last 60 s after equilibration. Anisotropy values were plotted as the change in anisotropy:

(1) $$\Delta 〈r〉(C)=〈r〉(C)-{〈r〉}_{0}$$

These values were used to calculate a bound fraction (*F*_bound_):

(2) $$F_{\text{bound}}(C)=\frac{\Delta 〈r〉(C)}{\Delta {〈r〉}_{\text{max}}}$$

The bound fraction was further used to calculate a dissociation constant (*K*_*d*_) by fitting to

(3) $$F_{\text{bound}}\left(C\right)=\frac{C}{C+K_{d}}$$

### Fluorescence anisotropy competition assays.

Competition assays were performed using the same measurement techniques described above for binding assays. In this experiment, 50 pmol of PosBD was added from DMSO into 125 μl of modified SELEX buffer, and the anisotropy was recorded for 2 min. One thousand picomoles of aptamer was then added and allowed to equilibrate for 10 min. After this time, 1,000 pmol of an unlabeled drug molecule was added, and the solution was heated up to 70°C for 3 min and cooled on ice for 2 min. The heat-ice cycle was performed twice. The anisotropy was then recorded again for another 5 min. The percentage of PosBD replaced was calculated as

(4) $$\%\text{ replaced}=100\times \frac{{〈r〉}_{\text{aptamer, drug}}-{〈r〉}_{0}}{{〈r〉}_{\text{aptamer}}-{〈r〉}_{0}}$$

The percentage replaced equals 100 multiplied by the anisotropy with aptamer and drug replacement minus the initial anisotropy divided by the anisotropy caused by the aptamer alone minus the initial anisotropy.

### CD experiments.

Experiments were performed using an Aviv model 420 CD spectrophotometer. All aptamer samples were prepared at a 10 μM concentration in 20 mM Tris buffer (pH 7.4). Increasing amounts of salts and/or azole antifungal drugs were added, and the CD spectra were recorded from 300 nm to 200 nm.

### GFET functionalization and testing.

The chemical vapor deposition (CVD) method with a methane source was used to grow graphene, which was then transferred via electrolysis onto a patterned Si/SiO_2_ surface. This surface contained chromium and gold electrodes, and the graphene channels between them were further defined through photolithography and annealed in an argon-hydrogen environment. GFETs were incubated in 1-pyrenebutyric acid *N*-hydroxysuccinimide ester (P-base) and dimethylformamide for 20 h. After this incubation, the devices were further incubated in a solution of phosphate-buffered saline (PBS; pH 7.6) containing the Rd 13 aptamer for 3 h. The devices were heated from 70 to 90°C, held at this temperature for 15 min, and then allowed to cool down to room temperature. In order to test the devices, the *I*-*V*_*g*_ properties (ideal transistor’s current-to-gate voltage) of GFETs were determined with posaconazole and caspofungin.
